# Dr. Addison and the First Council

**Published:** 1920-01-17

**Authors:** 


### THE MATRONS' AND SISTERS' DEPARTMENT.

### DR. ADDISON AND THE FIRST COUNCIL.

The Minister of Health may be said to hold the future of the nursing profession in the hollow of his hand. Parliament has given him a blank cheque, and the moment has arrived for him to fill in the figures. It is a matter of good augury that the Minister of Health has definitely taken a stand against the principle of representation by nomination. He does not believe that a homogeneous body can be brought into being by allowing groups of persons who have formed themselves into societies, as it were on their own initiative, to choose one out of their number to exercise the functions of councillor. The road to unity does not lie in that direction. The nomination system does in fact confer a premium on disunion, for every little separate group is claiming the right of nomination while the main body of nurses numbering matrons, sisters, and nurses from every training-school in the kingdom, having secured unity in the College of Nursing, are to be levelled to the mere status of yet another group. In fact, the only reasonably fair course to pursue if representation by nomination were to be adopted would be to confer on every local centre of the College the right of nominating a representative, and even then the membership of many of the centres would far outnumber that of other little groups which clamour for the privilege of separate nomination.

Dr. Addison plainly stated that he will have none of this competition for nomination. Its effect is to produce a heated and highly controversial atmosphere. The tendency is for persons to procure nomination not on account of their knowledge and powers, but because of their spirit of partisanship. The system has seldom produced a body commanding general approval, and it is capable of very mischievous manipulation. The Minister of Health told those who have been building on the chance of securing a majority on the Council by this method that he would not pledge himself to accept the nominations made by various bodies, and that he would not pledge himself not to go outside them if it were required. He wants to get a great work accomplished. A great increase of nursing facilities is required throughout the country and especially in rural areas, and we welcome the note in Dr. Addison's statements which tells of a forward policy.

What qualifications after all are most necessary in the first councillors whose duty it will be to lay the foundations of the new nursing profession deep and broad? The Minister of Health has himself told us. The first essential is that the council shall understand its business. He does not want an ornamental council, composed of persons whose names have been continually in the newspapers. He wants a working council, composed of people who have been through the mill. It is most necessary to remember that the field to take Council members from is the "mill" of to-day, not the "mill" of thirty or even twenty years ago. An immense wave of change has come over the nursing world. The simple methods which sufficed in olden days have given place to bewildering sub-division, specialisation, supplementary forms of training. Everyone knows that nurses of a past generation are of no use to a modern surgeon. It is equally true that the matrons of a past *régime* are not competent to direct nursing education to-day. It is not too much to say that no matron, however imposing her past experience, who has not had inside knowledge, at least within the last ten or fifteen years, of the working of the modern training-school system can be of any service in the great work of reconstruction and nursing reform which we await from the First Council.

Next in importance after first-hand knowledge of modern nursing among the qualifications needed in the Nursing Council we would place the ability to work harmoniously with colleagues, to accept defeat good-humouredly, to give opponents credit for good motives, to command respect and be content with the second best place. In selecting his councillors Dr. Addison will have the record of their work before him. He is not plunging into an unknown sea. The danger points are marked—the shoals and rocks which other generations of nurses have encountered. It is always difficult to act as impartial judge in bygone controversies. But in nursing matters the issue can be narrowed down to a fine point. It is possible to form a council absolutely free from the spirit of contention or personal antagonism, whether on one side or the other. The page on which the history of nursing reform is to be written is blank. Let not the pen fall into the hands of any who have blotted its past records. The great need of the moment is for reconciliation and unity. Only those, who have shown that they can sink self in the service of their profession, are worthy of a place on the First Council. Those whose names are identified with strife, yet, even if it be believed they fought in a good cause, are not suited for the searching collaboration which is to usher in the new era.

